# Detection of Circulating Serum Protein Biomarkers of Non-Muscle Invasive Bladder Cancer after Protein Corona-Silver Nanoparticles Analysis by SWATH-MS

**DOI:** 10.3390/nano11092384

**Published:** 2021-09-13

**Authors:** Benito Blanco Gómez, Rubén López-Cortés, Francisco Javier Casas-Nebra, Sergio Vázquez-Estévez, Daniel Pérez-Fentes, María del Pilar Chantada-Vázquez, Susana B. Bravo, Cristina Núñez

**Affiliations:** 1 Urology Division, Lucus Augusti University Hospital (HULA), Servizo Galego de Saúde (SERGAS), ES27002 Lugo, Spain; vvlanco@hotmail.com (B.B.G.); Francisco.Javier.Casas.Nebra@sergas.es (F.J.C.-N.); 2 Research Unit, Lucus Augusti University Hospital (HULA), Servizo Galego de Saúde (SERGAS), ES27002 Lugo, Spain; rlcortes.eu@gmail.com; 3 Oncology Division, Lucus Augusti University Hospital (HULA), Servizo Galego de Saúde (SERGAS), ES27002 Lugo, Spain; Sergio.Vazquez.Estevez@sergas.es; 4 Urology Division, University Clinical Hospital of Santiago de Compostela (CHUS), Servizo Galego de Saúde (SERGAS), ES15706 Santiago de Compostela, Spain; Daniel.Adolfo.Perez.Fentes@sergas.es; 5 Proteomic Unit, Health Research Institute of Santiago de Compostela (IDIS), University Clinical Hospital of Santiago de Compostela (CHUS), ES15706 Santiago de Compostela, Spain

**Keywords:** protein corona (PC), silver nanoparticles (AgNPs), bladder cancer (BC), biomarkers, fingerprinting, SWATH-MS

## Abstract

Because cystoscopy is expensive and invasive, a new method of detecting non-invasive muscular bladder cancer (NMIBC) is needed. This study aims to identify potential serum protein markers for NMIBC to improve diagnosis and to find treatment approaches that avoid disease progression to a life-threatening phenotype (muscle-invasive bladder cancer, MIBC). Here, silver nanoparticles (AgNPs, 9.73 ± 1.70 nm) as a scavenging device together with sequential window acquisition of all theoretical mass spectra (SWATH-MS) were used to quantitatively analyze the blood serum protein alterations in two NMIBC subtypes, T1 and Ta, and they were compared to normal samples (HC). NMIBC’s analysis of serum samples identified three major groups of proteins, the relative content of which is different from the HC content: proteins implicated in the complement and coagulation cascade pathways and apolipoproteins. In conclusion, many biomarker proteins were identified that merit further examination to validate their useful significance and utility within the clinical management of NMIBC patients.

## 1. Introduction

Bladder cancer (BC) is the second most common cancer of the genitourinary tract and one of the leading causes of death worldwide [1]. BC manifests as either muscle-invasive (MIBC) or non-muscle-invasive (NMIBC), the latter being the predominant form, comprising about 80% of cases [2]. Because 10-year recurrence rates of NMIBC are as high as 74.3%, cystoscopy is recommended routinely for surveillance, which contributes to increased expense and a higher risk for other urologic diseases in the patient [3,4]. Therefore, there is an urgent need of biomarkers for early screening of NMIBC, because they can promote early diagnosis, prevent the disease from developing into a life-threatening phenotype (MIBC), and ultimately reduce related treatment costs [5]. 

It is well known that proteins are the main regulators of cell functions, so proteomics characteristics will clearly reveal the pathological and physiological changes that occur during the development of carcinogenesis [6,7]. Blood samples, including plasma and serum, are considered to be a useful source for the development of proteomics methods and the discovery of non-invasive biomarkers because they are easily obtained from the blood and contain large amounts of abnormal proteins that can be used in circulation as tumors biomarkers [8]. 

Matrix-assisted laser desorption ionization with time-of-flight mass spectrometer (MALDI-TOF/MS), electrospray ionization with triple quadrupole mass spectrometer (ESI-QqQ/MS) and two-dimensional gel electrophoresis (2D-GE) combined with mass spectrometry (MS) were used to identify differentially expressed circulating proteins with high specificity and sensitivity [9,10,11]. In the case of BC, circulating prognosis and/or diagnosis biomarkers were recently discovered by liquid chromatography/mass spectrometry (LC-MS) [12,13,14]. However, there is still a relative lack of complete studies to examine the differently expressed proteins landscape in NMIBC. In this way, 2D-GE combined with MS were used to discover potential plasma biomarkers of NMIBC, and to identify variations between the proteome profiles of a cohort of patients with low-grade NMIBC and HC [15].

Despite these advances, there is an imperative to create strong high-throughput proteomic platforms for the identification of circulating proteins able to improve diagnosis and prognosis in the monitoring of the response to therapy in cancer [16,17,18]. In this manner, the recent feature in biomarker discovery is an emerging technique named SWATH-MS, also described as a liquid chromatography-tandem mass spectrometry (LC-MS/MS) based label-free quantitative proteomic platform [19]. Basically, SWATH-MS analysis has many advantages, including the discovery of high reproducibility and consistency of quantitative data in proteomics [20]. In addition, SWATH-MS is an important tool for discovering biomarkers and conducting preliminary validation studies [21].

Although blood is one of the most valuable biological fluids for the discovery of protein biomarkers, due to the wide dynamic range of blood protein concentration, it is difficult to use MS-based proteomics methods to identify these circulating biomarkers [6]. Furthermore, the ‘swamping’ effect caused by non-specific highly abundant proteins and also the low concentration of the biomarker proteins in biofluids, makes their detection by the current proteomic techniques difficult [22].

Recently, nanotechnology was applied to address the problems related to the discovery of protein biomarkers in biofluids. Therefore, since nanomaterials will form protein coronas (PCs) covering the nanomaterials when they are in contact with liquid biological media, nanoparticles (NPs) are ideal absorbent materials for enriching low-content peptides/proteins before subsequent processing by MS [23]. The PC acts as a “nano-concentrator” of serum proteins having an affinity for the surface of NPs. Therefore, the characterization of PC can reveal changes in protein levels that cannot be detected in the early stages of the disease, after chemotherapy or surgery [24]. Consequently, the same nanomaterial incubated with serum proteins from patients with completely different pathologies adsorbs PCs with different compositions, thus creating the new concept of personalized corona protein (PPC) [25].

Our previous reports collect robust protocols to the in vitro PC formation around gold nanoparticles (AuNPs, 10.02 ± 0.91 nm), silver nanoparticles (AgNPs, 9.73 ± 1.70 nm), platinum nanoparticles (PtNPs, 2.40 ± 0.30 nm) and magnetic nanoparticles (MNPs, 9.30 ± 0.67 nm) after their interaction with human serum (HS) [26,27]. The combination of these nanomaterials with electrophoretic separation (SDS-PAGE) and LC-MS/MS allowed the discovery of novel serum protein biomarkers of triple-negative breast cancer (TNBC) [27]. Importantly, PC-AuNP fingerprinting revealed a profile of neutrophil-derived granule proteins in the serum of breast cancer patients [28] and, in particular, a profile of blood coagulation proteins in the serum of HER2-overexpressing breast cancer patients [29]. Molecular targets associated with thyroid tumors were also identified after the application of a similar approach for the analysis of protein extracts from thyroid tissue samples [30]. 

**Figure 1:**
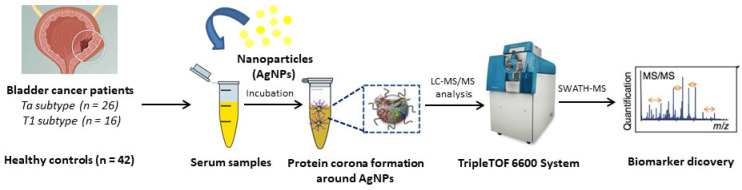
Schematic representation of the methodology developed for the PC formation around AgNPs (9.73 ± 1.70 nm) after the ex vivo incubation with HS samples from HC (*n* = 42) and NMIBC patients (*n* = 42). The quantitative analysis of the PCs was developed by SWATH-MS for the detection of circulating protein biomarkers.

Although we have previously identified new targets after analyzing the PC formed around AgNPs (with strong antibacterial) in contact with biofluids [26,27], to the best of our knowledge this is the first work in which a nanoparticle-assisted proteomics approach based on AgNPs-PC fingerprinting combined with shotgun LC-MS/MS analysis (SWATH-MS) were developed for the identification of novel targets in serum samples from NMBC patients (see Figure 1).

## 2. Materials and Methods

### 2.1. Chemicals 

All reagents and solvents used were HPLC/LC-MS or electrophoresis grade. Ammonium bicarbonate (AMBIC, 99.5%); ammonium persulfate (APS, 98%); β-mercaptoethanol (99%); glycerol (86–88%); silver nitrate (99%); sodium carbonate (99%); sodium citrate tribasic dihydrate (99%); tannic acid; trifluoroacetic acid (TFA, 99%); tris-base; trizma base (99.9%); trypsin from bovine pancreas and urea were purchased from Merck (Barcelona, Spain). Acrylamide/bis-acrylamide 30% solution (37.5:1) were purchased from Serva (Heidelberg, Germany). Bromophenol-blue; CCB: Coomassie Brilliant Blue R250 staining solution; DL-dithiothreitol (DTT); iodoacetamide (IAA, 99%); sodium dodecylsulfate (SDS); TEMED and the molecular wide range scale marker (mol wt 6.5–200 kDa) for SDS-PAGE were purchased from Bio-Rad (Madrid, Spain). Solvents were supplied by Panreac Química SLU (Barcelona, Spain).

### 2.2. Biological Samples

From January 2018 to June 2019, peripheral venous blood samples from 42 NMIBC patients (age varying from 31 to 86 years) and from 42 age-matched healthy controls (HC) were collected at HULA from Lugo (Spain). In this work, NMIBC patients (*n* = 42) were categorized into two subtypes: Ta subtype (*n* = 26) and T1 subtype (*n* = 16) and samples were collected before they underwent surgery and/or before receiving any treatment. 

All blood samples were collected in VACUETTE^®^ Serum Clot Activator Tubes (9 mL), allowed to clot for 15 min, and centrifuged at 1800× *g* (5 min, 4 °C). The resultant serum was transferred to sterile cryovials, frozen, and stored at −80 °C until further use.

The study was approved by the Clinical Research Ethics Committees (CEIC) of Galicia (Spain) (number 2017/419) and it had been conducted in conformity with the declaration of Helsinki. Furthermore, all participants provided written consent before their participation within the study.

### 2.3. Synthesis of Citrate-Silver Nanoparticles (AgNPs, 9.73 ± 1.70 nm) and Ex Vivo Protein Corona Formation

**Figure 2:**
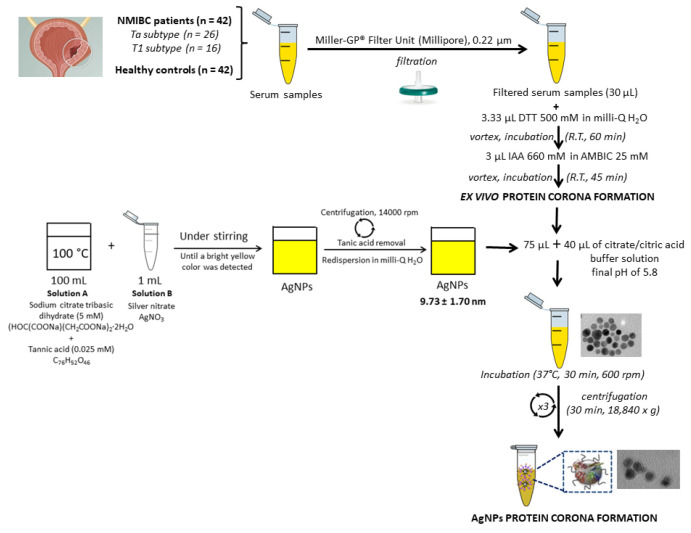
Flowchart depicting the synthesis of AgNPs (9.73 ± 1.70 nm), the pretreatmen of HS samples and PCs formation.

AgNPs were synthesized by a citrate reduction method [31] consisting of the addition of 1 mL of AgNO_3_ (25 mM) to an aqueous boiling solution (100 mL) of sodium citrate (SC) (5 mM) and tannic acid (TA) (0.025 mM). The resultant solution was kept under heating and vigorous stirring until a bright yellow color was observed which indicated the formation of well-dispersed AgNPs. Excess of SD and TA was removed by centrifugation at 18,840× *g* (×3) for 30 min. Resultant pure AgNPs were further redispersed in Milli-Q-water and the formation of the ex vivo PC was achieved following the steps shown in Figure 2 [26,27,28,29]. The characterization of the colloidal AgNPs and the formation of the PCs were confirmed by transmission electron microscopy (TEM) on a JEM 1011, JEOL instrument (Santiago de Compostela, Spain). The ζ-potentials of colloidal AgNPs were measured (×3) with a Malvern Zetasizer Nano ZS Instrument (Santiago de Compostela, Spain) at 25 °C. The total amount of the proteins adsorbed onto the surface of AgNPs was quantified by using a Qubit™ 4 Quantitation Starter Kit (Thermo Fisher Scientific, Waltham, MA, USA) according to the manufacturer’s instructions. Protein binding (Pb) values, expressed as μg of protein per μmole of AgNPs were then calculated per patient and per group, presented as the average ± standard deviation (*n* = 42 HC and *n* = 42 NMIBC patients), following a previously reported method [32].

### 2.4. Quantification of the Proteins Presented in the Corona-Coated AgNPs by SWATH-MS 

Corona proteins associated with AgNPs were separated by SDS-PAGE using a PowerPac^TM^ Basic Power Supply (Bio-Rad, Madrid, Spain). After that, corona proteins were digested following the method previously described in detail [27,29].

SWATH-MS acquisition and processing of the data were as previously stated, with slight modifications [27,29]. Briefly, a spectral library was first created using pooled samples from each group (HC, NMIBC with the T1 subtype, NMIBC with the Ta subtype) via a shotgun data-dependent acquisition (DDA) approach by micro-LC. Then, an MS/MSALL add-in for PeakView Software (v. 2.2., Sciex, Redwood City, CA, USA) was employed for the peak extraction using the SWATH Acquisition MicroApp (v. 2.0., Sciex, Redwood City, CA, USA). The construction of the spectral library only included peptides with a confidence score >99%. SWATH–MS acquisition was performed on a TripleTOF^®^ 6600 LC-MS/MS system (Sciex, Redwood City, CA, USA) via a data-independent acquisition (DIA) method. Extraction of peaks and the relative quantitative analysis were performed in the PeakView (v. 2.2) using the SWATH Acquisition MicroApp (v. 2.0) and the MarkerView software (Sciex, Redwood City, CA, USA), respectively. The integrated peak areas (processed mrkvw files from PeakView) were directly exported to MarkerView software (Sciex, Redwood City, CA, USA) for relative quantitative analysis. The average MS peak area for each protein was derived from the biological replicates of the SWATH-MS of each sample, followed by analysis using a Student’s *t*-test (MarkerView software, sciex, Redwood City, CA, USA) to compare between samples based on the averaged total area of all transitions for each protein. The *t*-test result (*p*-value) indicates how well each variable distinguishes the two groups. Candidate proteins were selected for each library based on *t*-test results (*p*-value < 0.05 and FC (increase or decrease) > 1.4).

### 2.5. Protein Functional Interaction Network Analysis 

The analysis of the networks of functional interaction of proteins, integrating the direct (physical) and indirect protein-protein interactions (PPI) was carried out with the tool STRING v.10.0 database (http://string-db.org (accessed on 15 January 2021)) [33]. 

## 3. Results and Discussion 

### 3.1. Incubation of AgNPs (9.73 ± 1.70 nm) with HS Samples: Ex Vivo Protein Corona Formation and Characterization

HS samples from *n* = 42 HC and *n* = 42 NMIBC patients were collected, processed, and analyzed in the same manner (see Section 2.2). The NMIBC patient group was divided into the following biological subtypes: *n* = 26 patients with Ta subtype and *n* = 16 patients with T1 subtype. 

AgNPs with a size of 9.73 ± 1.70 nm were prepared by a chemical reduction method [26,27,28,29]. As shown in Figure 2, proteins contained in serum samples (x2) were chemically reduced with DTT and alkylated with IAA before incubating with AgNPs (9.73 ± 1.70 nm) to get the formation of the PCs [26,27,28,29]. 

**Figure 3:**
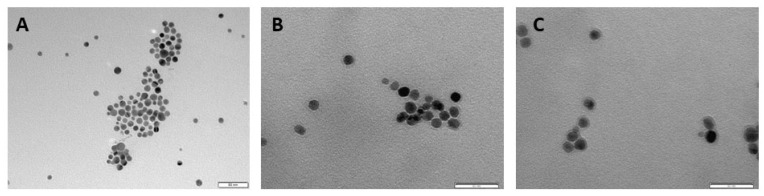
Negative stain transmission electron microscopy (TEM) imaging of bare AgNPs before (**A**) and after the ex vivo incubation with a pool of serum samples of HC (*n* = 42) (**B**) and pool of HS samples of NMIBC patients (*n* = 42) (**C**), resulting in the formation of the PCs.

After incubating AgNPs with a pool of HS samples from HC (*n* = 42) and pool of HS samples from NMIBC patients (*n* = 42), the resulting PC-coated AgNPs were centrifuged and characterized by TEM and dynamic light scattering (DLS). The interaction between serum proteins and AgNPs surface caused an increase of the size of the AgNPs, from 9.73 ± 1.70 nm to 11.91 ± 1.37 nm (HC) and 13.38 ± 1.93 nm (NMIBC) (see Tables S1–S3). The preferred interaction of the positively charged proteins with the AgNPs surface may cause the average surface charge of the NPs to increase from −40.5 mV (bare AgNPs) to −28.6 mV (HC) and −26.6 mV (NMIBC) [34,35]. TEM images show a large number of well-dispersed NPs, confirming the formation of PCs around AgNPs (see Figure 3). 

To quantitatively compare the total amount of protein adhered onto AgNPs in the two different conditions under investigation (HC and NMIBC patients), the protein binding value (Pb), expressed as the total amount of protein (in μg) per μmole of AgNPs, was estimated. The total amount of proteins adsorbed onto AgNPs after their incubation with HS samples obtained from NMIBC patients (Pb = 52 μg of protein/μmole of AgNPs) was similar to the amount of proteins adsorbed onto AgNPs after their incubation with HS obtained from HC (Pb = 46 μg of protein/μmole of AgNPs).

### 3.2. Quantitative Analysis of the Protein Corona-Coated AgNPs by SWATH-MS

Corona proteins associated with AgNPs were separated by SDS-PAGE and digested following a previously reported method [26,27,28,29]. The resulting peptides were then quantitative analysed by the emerging proteomic platform for label-free quantification SWATH-MS.

The comparison of the ex vivo PCs patterns makes it possible to identify proteins that express differently between HC and the two NMIBC subtypes (Ta and T1). Results were filtered to present a *p*-value ≤ 0.05 and interestingly, *n* = 40 proteins were found to be differentially expressed, of which *n* = 11 were up-regulated and *n* = 29 down-regulated in NMIBC patients with the T1 subtype, and *n* = 46 proteins were found to be differentially expressed, of which *n* = 30 were up-regulated and *n* = 16 down-regulated in NMIBC patients with the Ta subtype (see Table 1). The full list of candidate protein biomarkers identified to be up-regulated or down-regulated in both NMIBC subtypes in comparison to HC with the fold-change values is shown in Table S4.

**Figure 4:**
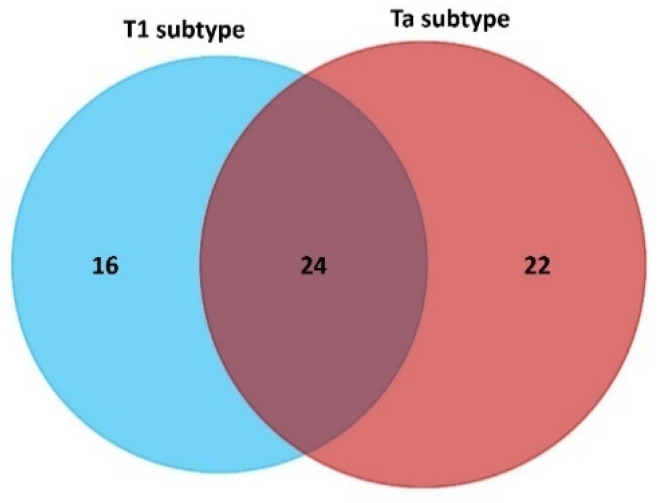
Venn diagram showing the number of shared and specific deregulated proteins identified in the PCs coated AgNPs (9.73 ± 1.70 nm) after their incubation (30 min) with HS samples from NMIBC patients (subtypes T1 and Ta).

The Venn diagram of statistically significant up- and down-regulated proteins shows that 24 proteins were found to be commonly altered in the T1 and Ta subtypes (see Figure 4). Particularly, 10 of these 24 proteins were found to be up-regulated in both NMIBC subtypes: thrombospondin-1 (THBS1), apolipoprotein C-II (APOC2) plasminogen (PLG), serum amyloid A-4 protein (SAA4), plasma kallikrein (KLKB1), lipopolysaccharide-binding protein (LBP), complement C1r subcomponent (C1R), complement C1s subcomponent (C1S), coagulation factor IX (F9), coagulation factor XII (F12). Furthermore, 14 of these 24 proteins were found to be down-regulated in both NMIBC subtypes: carboxypeptidase N catalytic chain (CPN1), carboxypeptidase N subunit 2 (CPN2), apolipoprotein M (APOM), insulin-like growth factor-binding protein complex acid labile subunit (IGFALS), alpha-2-HS-glycoprotein (AHSG), fibronectin (FN1), serum paraoxonase/arylesterase 1 (PON1), plasma protease C1 inhibitor (SERPING1), mannan-binding lectin serine protease 1 (MASP1), complement C2 (C2), sex hormone-binding globulin (SHBG), N-acetylmuramoyl-L-alanine amidase (PGLYRP2), antithrombin-III (SERPINC1), prothrombin (F2) (Table S4).

SWATH-MS was also used to identify subtype-specific proteins (see Figure 4 and Table 2) for the T1 subtype (*n* = 16 specific proteins: *n* = 1 upregulated and *n* = 15 downregulated) and the Ta subtype (*n* = 22 specific proteins: *n* = 20 upregulated and *n* = 2 downregulated).

### 3.3. The Biological Role of the NMIBC-Related Proteins Identified in the AgNPs-Protein Corona 

To interpret global changes in the serum proteome associated with NMIBC, the 62 proteins (16 specific of T1, 22 specific of Ta, 24 commons of T1 and Ta) whose levels were significantly different between HC and NMIBC patients were analyzed using the STRING software. The analysis revealed that two pathways were mainly associated with 29 of 62 dysregulated serum proteins, including the complement and coagulation cascades pathways. These biomarker proteins are subdivided in:

(a) complements: C1r subcomponent (C1R), complement C1s subcomponent (C1S), complement C2 (C2), complement C4-A (C4A), complement C4-B (C4B), C4b-binding protein alpha chain (C4BPA), complement C5 (C5), complement component C6 (C6), complement component C7 (C7), complement component C8 beta chain (C8B), complement component C8 gamma chain (C8G), complement component C9 (C9), complement factor B (CFB), complement factor H (CFH), and complement factor I (CFI);
(b) coagulation factors: coagulation factor II (F2), coagulation factor IX (F9) coagulation factor (F10) and coagulation factor XII (F12);
(c) serine protease related proteins: alpha-1-antitrypsin (SERPINA1), antithrombin-III (SERPINC1), alpha-2-antiplasmin (SERPINF2), plasma protease C1 inhibitor (SERPING1), plasma kallikrein (KLKB1) and plasminogen (PLG);
(d) vitamin K-dependent proteins: vitamin K-dependent protein C (PROC) and vitamin K-dependent protein S (PROS1); and
(e) glycoproteins: fibrinogen alpha chain (FGA) and vitronectin (VTN).

**Figure 5:**
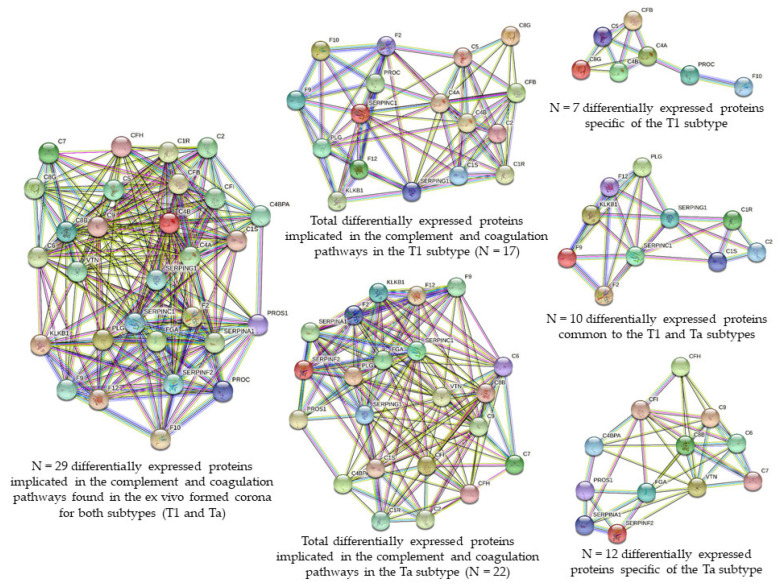
Clusters found in the protein–protein interaction network map of the 62 genes encoded differentially proteins identified in the PC coated AgNPs (9.73 ± 1.70 nm) after their incubation (30 min) with HS samples from 42 NMIBC patients. Based on the STRING database, 29 differential expressed proteins formed a network core with proteins implicated in the complement and coagulation cascades, from them 10 proteins were commonly found to be deregulated in the T1 and the Ta subtypes, seven proteins were specific of the T1 subtype and 12 proteins were specific of the Ta subtype.

From these 29 proteins involved in the complement and coagulation cascades, 17 and 22 were found to be related to the T1 and the Ta subtypes, respectively. However, while 10 of these proteins were commonly found to be deregulated in the T1 and the Ta subtypes (down-regulated: F2, C2, SERPINC1, SERPING1; up-regulated: C1S, C1R, F9, F12, KLKB1, PLG), seven proteins were specific of the T1 subtype (C4A, C4B, C5, C8G, CFB, F10, PROC, all down-regulated) and 12 proteins were specific of the Ta subtype (down-regulated: SERPINF2, PROS1; up-regulated: C4BPA, C6, C7, C8B, C9, CFH, CFI, SERPINA1, FGA, VTN) (see Figure 5, Table 2 and Table S1).

The complement system is a cascade of serine proteases encoded by the same genes as coagulation proteins [36]. Like the coagulation system, complement activation is highly regulated and involves multiple stages that require plasma and membrane proteins [37,38]. The complement system is part of the innate and ancient defenses against pathogen invasion. It is also involved in inflammation, adaptive immune response, hemostasis, embryogenesis, and organ repair and development. Activation of the complement system through classical, lectin, or alternative pathways produces various compounds, such as anaphylatoxins (C3a and C5a) and membrane attack complex (C5b-9), and regulates targeted cells. The end products of complement system and its receptor activation mediate cell-cell interactions, thereby regulating various biological pathways in extravascular tissues. The assembly of membrane attack complexes and signal transmission through the anaphylatoxin receptor stimulate dedifferentiation, cell proliferation and migration and reduce cell apoptosis. Therefore, the activation of the complement system in the tumor microenvironment promotes tumor growth and metastasis. The higher expression of complement-derived products in cancer cell lines and malignant tumors has been described [39]. Thus, based on the complement activation within tumors, it is easy to detect the degradation products of C3, complement proteins, and complement activation products (i.e., C5a, C3a, and C5b-9) in various cancers. The main pathway involved in complement activation in tumors are not yet clear, and data support the activation of various complement pathways in malignant tumors [40]. 

In the particular case of BC, Chien-Lun Chen et al. [41] developed an iTRAQ study where they also observed that complement and blood-coagulation pathways were likely stimulated for the duration of bladder tumor development and invasion. In particular, they found increased levels of proteins associated with both pathways after the analysis of the BC urine proteome, as plasminogen (PLG) and plasma kallikrein (KLKB1). This study shows that complement and coagulation pathways are likely to establish models in the development and invasion of bladder tumors. The proteins involved in these signaling pathways may be secreted in urine, that is, in the proximal bladder fluid, and their levels in the BC urine proteome are elevated. In the present work, PLG and KLKB1 proteins were also found to be up-regulated in the T1 and Ta subtypes. 

In 2020, T. Nedjadi et al. [15] analyzed plasma samples from patients with low-grade NMIBC and HC using combined 2D-DIGE and mass-spectrometry and they found that complement C1r subcomponent (C1R) was significantly up-regulated in tumors compared to controls. Furthermore, complement factor H (CFH) was also previously reported to be a marker for the detection of BC [42]. In the present work, while C1R was also found to be up-regulated in T1 and Ta subtypes, CFH were found to be up-regulated in the Ta subtype. 

**Figure 6:**
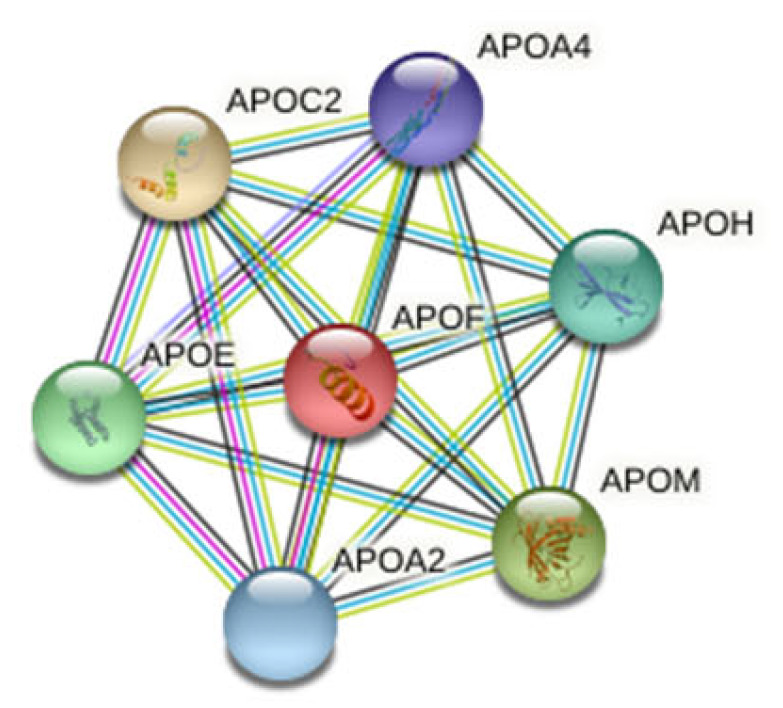
Cluster found in the protein–protein interaction network map of the 62 genes encoded differentially proteins identified in the PC coated AgNP (9.73 ± 1.70 nm) after their incubation (30 min) with HS samples from 42 NMIBC patients. Based on the STRING database, seven differential expressed proteins formed a network core with proteins implicated in the lipid transport.

Furthermore, in the present work, 7 of the 62 tumor-related proteins involved in lipid transport were identified (see Figure 6). While apolipoprotein C-II (APOC2) was found to be up-regulated in the T1 and Ta NMIBC subtypes, apolipoprotein M (APOM) were found to be down-regulated in both subtypes. However, most apolipoproteins were specific of the subtype T1: (a) down-regulated: apolipoprotein A-II (APOA2), apolipoprotein A-IV (APOA4), apolipoprotein E (APOE) and apolipoprotein F (APOF); (b) up-regulated: apolipoprotein H (APOH), also knows as beta-2 glycoprotein 1. 

The diagnostic potential of some of these apolipoproteins in the detection of different cancers were previously demonstrated, particularly in BC [43]. For example, APOE in the urine of BC patients is significantly increased, and its value is related to tumor stage [44]. Thus, APOE testing of the urine could provide a potential marker for NMIBC [43]. Lindén et al. reported that as part of a panel of biomarkers, APOE detected by MS and validated by Western blot analysis may be also valuable for the detection of NMIBC [45]. Although in the present work APOE was found to be down-regulated in the serum of NMIBC patients with the T1 subtype, possible mechanisms underlying the APOE role in cancer include increased cell proliferation and enhanced lipid transport into tumor cells [46,47]. In particular, in BC the reaction of the transitional epithelial response gene TERE1 (a tumor suppressor gene) with APOE resulted in an increase of the cell turnover and a resistance to apoptosis [48,49]. 

As mentioned above, APOC2 was found to be up-regulated in both NMIBC subtypes, T1 and Ta. Similarly, serum levels of APOC2 were also found to be elevated in pancreatic cancer patients compared to controls [50]. 

Similar to the results observed for the T1 subtypes, the serum level of APOA2 was also significantly reduced in patients with multiple myeloma gastric cancer [51,52], and in plasma of patients with pancreatic cancer [53]. In addition, the level of APOA4 in the serum of patients with ovarian cancer was also found to be decreased [54]. According to reports, overexpression of APOM can inhibit the migration, proliferation and invasion of liver cancer cells and the development of xenograft tumors in nude mice, thereby inducing cell apoptosis [55]. The down-regulation observed in the T1 and Ta subytpes, was also observed in human hepatocellular carcinoma tissues compared to adjacent healthy tissues [56], as well as in colorectal cancer tissues, compared to adjacent healthy tissues, polyps, normal mucosa and inflammatory mucosa [57].

For example, other apolipoproteins, such as serum amyloid A-4 (SAA4), have a three-fold increase in urine levels in kidney and BC samples [41]. In addition, the degree of increase in SAA4 levels is related to the disease state of BC patients, which indicates the potential use of urine SAA4 levels as a degree discriminator. These findings suggest that urinary SAA4 is a singular biomarker for detecting urological derived tumors. The same authors also found that six apolipoproteins (APOA1, APOA2, APOB, APOC2, APOC3, and APOE) can distinguish BC from controls and are statistically significant [41]. In a similar manner, SAA4 was also found to be up-regulated in the serum of NMIBC patients with two subtypes (Ta and T1) in the present study. 

Finally, the current work identified other previously described biomarkers related to BC, such as vitamin D-binding protein (GC) and pigment epithelium-derived factor (PEDF), which are up-regulated in the Ta subtype and down-regulated in the T1 subtype, respectively. A recent study by M. Ożgoet al. [58] also revealed that the relative GC content in plasma samples of bladder urothelial patients was eight times higher than that in normal plasma samples, indicating that the protein was significantly related to BC. However, the role of GC in the carcinogenic process of BC is controversial and requires further analysis [59]. On the other hand, is is reported that PEDF is one of the most powerful natural anti-angiogenic factors, which can inhibit tumor growth in various forms of cancer [60,61,62,63], either by antagonizing tumor angiogenesis or exerting direct inhibition. In particular, a decreased PEDF expression in bladder tumors was previously reported [64] and was found to be related to differentiation, invasiveness, and angiogenesis of the bladder tumor [65]. Current results of the present work could be very valuable for the further development of targeted anti-angiogenic therapy against NMIBC.

**Figure 7:**
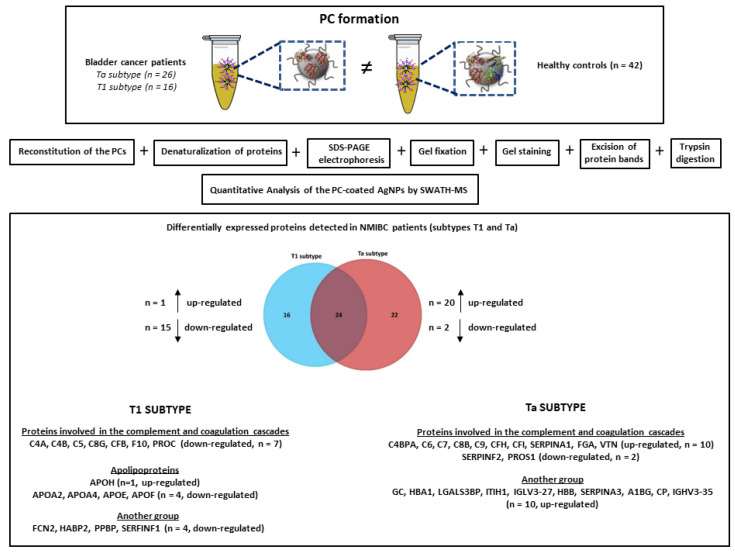
Illustration integrating the experimental procedure and the main results obtained in the present work.

A summary picture integrating all the above results is shown in Figure 7. 

Finally, many factors could affect the stability of AgNPs [66,67,68,69]. Of these, pH had a strong influence on the properties of the AgNPs, as it governed the surface charge of AgNPs hence aggregation and oxidative dissolution [70]. In particular, it was found that at acidic and neutral pH, AgNPs were destabilized resulting in higher rate of aggregation, and in alkaline conditions, AgNPs were re-stabilized due to the presence of hydroxyl ions resulting in more stable suspensions [69]. In the present work, in the incubation of AgNPs with serum samples in slightly acidic conditions (pH value 5.8) could take place the aggregation and oxidative dissolution of the colloidal suspension. Thus, in future work, a basic pH could be more appropriated for the PC evaluation. 

Furthermore, it is important to mention that the PC formation is a continuous and competitive process, through dynamic interactions of proteins to the nanomaterial surface [71]. The composition changes over time by displacement of earlier adsorbed proteins (“soft corona”) by other proteins with stronger binding affinities (“hard corona”) until the equilibrium is reached (“Vroman effect”) [72,73,74,75,76]. The composition of the PC is affected by many factors, such as the surface characteristics, biological environment around NPs, times of exposure, and physiochemical properties (size and charge) of NPs [77,78,79,80,81]. Mainly, hydrophobicity of NPs may determine the nature of PC [82,83,84]. The main driving force for the PC formation may be the non-covalent, non-specific, and hydrophobic interactions between proteins and NPs, whose strength could be significantly affected by the media [85]. In the present work, to fully understand the mechanisms of interactions between the identified biomarkers proteins mentioned above and the hydrophobic AgNPs surface the three-dimensional structure of these proteins should be considered [86,87]. In further works, we will use computation modeling to investigate the related selectivity and affinity mechanisms. 

## 4. Conclusions

The clinical purpose of this study is to discover non-invasive stage/grade discriminators or predictors of tumor progression in BC. Quantitative comparison of the isolated PCs produced after the incubation of AgNPs with serum samples obtained from NMIBC patients revealed 62 deregulated subtype-specific proteins (16 and 22 proteins specifically related to T1 and Ta subtypes, respectively, and 24 proteins commonly altered in both subtypes). The ex vivo analysis of the PCs formed in AgNPs allowed us to distinguish two main groups of serum protein whose relative levels in NMIBC patients are different from those in normal samples: proteins involved in the complement pathway and coagulation cascade, and apolipoproteins. 

Additional information from studying these altered proteins as potential potentially powerful prognosis biomarkers and targets for new therapies may have a profound impact on the management of NMIBC patients. In particular, anti-complement and anticoagulant reagents can occupy a place in the anti-NMIBC therapeutic arsenal, and due to their non-overlapping pharmacodynamics and limited non-myelosuppressive side effects, they can be combined with conventional immunotherapy or chemotherapy.

## Figures and Tables

**Table 1:** The total amount of different expressed proteins (up-regulated and down-regulated, *p*-value ≤ 0.05) and specific to the different NMIBC subtypes (T1 and Ta) found after the SWATH-MS analysis of PC coated AgNPs (9.73 ± 1.70 nm).

SWATH-MS Analysis						
**Comparison**	**Protein Number (*p*-Value ≤ 0.05)**					
	**Total**	**Up-Regulated**	**Down-Regulated**	**Specific**	**Up-Regulated**	**Down-Regulated**
Controls vs. T1	40	11	29	16	1	15
Controls vs. Ta	46	30	16	22	20	2

Background blue color: data related to the T1 subtype. Background orange color: data related to the Ta subtype.

**Table 2:** Specific differentially expressed proteins detected in NMIBC patients (subtypes T1 and Ta) relative to the control group after the analysis of the PC coated AgNPs (9.73 ± 1.70 nm) by SWATH-MS. The fold change ratio was calculated as the ratio of the geometric mean of the samples, corresponding to the calculation of the normal arithmetic ratio of the logarithmic transformation and inverse transformation regions.

Protein Name	UniProt Name	Gene	*p*-Value	Fold Change	NMIBC Sybtype
Beta-2-glycoprotein 1	APOH_HUMAN	APOH	0.031038566	1.413139568	↑ T1
Complement C4-A	CO4A_HUMAN	C4A	3.35E-05	1.872021358	↓ T1
Apolipoprotein F	APOF_HUMAN	APOF	0.000241559	2.110329795	↓ T1
Complement factor B	CFAB_HUMAN	CFB	0.000636998	1.372510381	↓ T1
Complement C4-B	CO4B_HUMAN	C4B	0.001425038	1.622969299	↓ T1
Complement component C8 gamma chain	CO8G_HUMAN	C8G	0.003262992	1.467525612	↓ T1
Ficolin-2	FCN2_HUMAN	FCN2	0.003600193	1.765148338	↓ T1
Apolipoprotein E	APOE_HUMAN	APOE	0.004492395	1.342803692	↓ T1
Apolipoprotein A-IV	APOA4_HUMAN	APOA4	0.004832934	1.330273828	↓ T1
Hyaluronan-binding protein 2	HABP2_HUMAN	HABP2	0.005064575	1.385807696	↓ T1
Platelet basic protein	CXCL7_HUMAN	PPBP	0.007192858	1.607064055	↓ T1
Pigment epithelium-derived factor	PEDF_HUMAN	SERPINF1	0.0177288	2.054793133	↓ T1
Complement C5	CO5_HUMAN	C5	0.022678206	1.261047319	↓ T1
Vitamin K-dependent protein C	PROC_HUMAN	PROC	0.023388014	1.233676674	↓ T1
Coagulation factor X	FA10_HUMAN	F10	0.028581041	1.261414143	↓ T1
Apolipoprotein A-II	APOA2_HUMAN	APOA2	0.029458523	2.388772729	↓ T1
Fibrinogen alpha chain	FIBA_HUMAN	FGA	0.00018616	1.692142233	↑ Ta
Complement component C7	CO7_HUMAN	C7	0.0014036	1,37740437	↑ Ta
Vitronectin	VTNC_HUMAN	VTN	0.00148246	1.330829684	↑ Ta
Complement component C9	CO9_HUMAN	C9	0.00259827	1.326995622	↑ Ta
Vitamin D-binding protein	VTDB_HUMAN	GC	0.003928	1.340366003	↑ Ta
Complement component C6	CO6_HUMAN	C6	0.0042717	1.446886498	↑ Ta
Complement component C8 beta chain	CO8B_HUMAN	C8B	0.00538557	1.255996294	↑ Ta
Hemoglobin subunit alpha	HBA_HUMAN	HBA1	0.00590282	5.020407249	↑ Ta
Galectin-3-binding protein	LG3BP_HUMAN	LGALS3BP	0.00753614	2.016590504	↑ Ta
Inter-alpha-trypsin inhibitor heavy chain H1	ITIH1_HUMAN	ITIH1	0.00788349	0.719608487	↑ Ta
Complement factor I	CFAI_HUMAN	CFI	0.00810041	1.222227898	↑ Ta
Immunoglobulin lambda variable 3–27	LV327_HUMAN	IGLV3-27	0.01005932	1.783180848	↑ Ta
Hemoglobin subunit beta	HBB_HUMAN	HBB	0.01030231	4.673494773	↑ Ta
Alpha-1-antichymotrypsin	AACT_HUMAN	SERPINA3	0.01546691	5.505121842	↑ Ta
C4b-binding protein alpha chain	C4BPA_HUMAN	C4BPA	0.01766023	1.267495989	↑ Ta
Alpha-1B-glycoprotein	A1BG_HUMAN	A1BG	0.03046776	1.640307834	↑ Ta
Alpha-1-antitrypsin	A1AT_HUMAN	SERPINA1	0.03633694	3.239474829	↑ Ta
Ceruloplasmin	CERU_HUMAN	CP	0.03816982	1.652775639	↑ Ta
Immunoglobulin heavy variable 3-30-5	HV335_HUMAN	IGHV3-35	0.04091296	1.749806744	↑ Ta
Complement factor H	CFAH_HUMAN	CFH	0.04493203	1.213794163	↑ Ta
Vitamin K-dependent protein S	PROS_HUMAN	PROS1	0.00653335	1.241725216	↓ Ta
Alpha-2-antiplasmin	A2AP_HUMAN	SERPINF2	0.00979701	1.476811908	↓ Ta

Background blue color: data related to the T1 subtype. Background orange color: data related to the Ta subtype.

## Data Availability

The mass spectrometry proteomics data have been deposited to the ProteomeXchange Consortium via the PRIDE partner repository with the dataset identifier PXD028310.

## Supplementary Materials

The following are available online at https://www.mdpi.com/article/10.3390/nano11092384/s1, Table S1: TEM image and characterization data of AgNPs@citrate in Milli-Q-water. Table S2: TEM image and characterization data of AgNPs@PC-HC (healthy controls) in Milli-Q-water. Table S3: TEM image and characterization data of AgNPs@PC-NMIBC (patients) in Milli-Q-water. Table S4: Differentially expressed proteins (up- and down-regulated) (*p*-value ≤ 0.05) detected in NMIBC patients (subtypes T1 and Ta) relative to the control group after the analysis of the PC coated AgNPs (9.73 ± 1.70 nm) by SWATH-MS.
