# Supplementary material for: Detection of Circulating Serum Protein Biomarkers of Non-Muscle Invasive Bladder Cancer after Protein Corona-Silver Nanoparticles Analysis by SWATH-MS
Source: Nanomaterials (Basel). 2021 Sep 13;11(9):2384. doi: 10.3390/nano11092384 (PMC8467878; doi:10.3390/nano11092384)
Supplement: Supplementary file 1 [file nanomaterials-11-02384-s001.zip › nanomaterials-1330232-supplementary.pdf]

Supplementary Materials

Detection of Circulating Serum Protein Biomarkers of Non-Muscle Invasive Bladder Cancer after Protein Corona-Silver Nanoparticles Analysis by SWATH-MS

Benito Blanco Gómez [1], Rubén López-Cortés [2], Francisco Javier Casas-Nebra [1], Sergio Vázquez-Estévez [3], Daniel Pérez-Fentes [4], María del Pilar Chantada-Vázquez [2,5,*], Susana B. Bravo [5,*] and Cristina Núñez [2,*]

[1] Urology Division, Lucus Augusti University Hospital (HULA), Servizo Galego de Saúde (SERGAS), ES27002 Lugo, Spain; vvlanco@hotmail.com (B.B.G.); Francisco.Javier.Casas.Nebra@sergas.es (F.J.C.-N.)

[2] Research Unit, Lucus Augusti University Hospital (HULA), Servizo Galego de Saúde (SERGAS), ES27002 Lugo, Spain; rlcortes.eu@gmail.com

[3] Oncology Division, Lucus Augusti University Hospital (HULA), Servizo Galego de Saúde (SERGAS), ES27002 Lugo, Spain; Sergio.Vazquez.Estevez@sergas.es

[4] Urology Division, University Clinical Hospital of Santiago de Compostela (CHUS), Servizo Galego de Saúde (SERGAS), ES15706 Santiago de Compostela, Spain; Daniel.Adolfo.Perez.Fentes@sergas.es

[5] Proteomic Unit, Health Research Institute of Santiago de Compostela (IDIS), University Clinical Hospital of Santiago de Compostela (CHUS), ES15706 Santiago de Compostela, Spain

* Correspondence: mariadelpilarchantadavazquez@gmail.com (M.d.P.C.-V.); sbbravo@gmail.com (S.B.B.); cristina.nunez.gonzalez@sergas.es (C.N.)

Table S1. TEM image and characterization data of AgNPs@citrate in Milli-Q-water.

**AgNPs@citrate (nm)**			
1	7.58	21	9.30
2	7.66	22	9.45
3	7.89	23	9.58
4	7.89	24	9.76
5	7.89	25	9.86
6	7.98	26	10.25
7	8.17	27	10.39
8	8.19	28	10.48
9	8.19	29	10.61
10	8.21	30	10.99
11	8.40	31	11.17
12	8.40	32	11.27
13	8.48	33	11.47
14	8.55	34	11.64
15	8.64	35	11.83
16	8.75	36	11.83
17	8.92	37	12.10
18	9.02	38	13.02
19	9.07	39	13.49
20	9.16	40	13.76
Count	40		
Mean	9.73		
Minimum	7.58		
Maximum	13.76		
Standar Deviation	1.70		

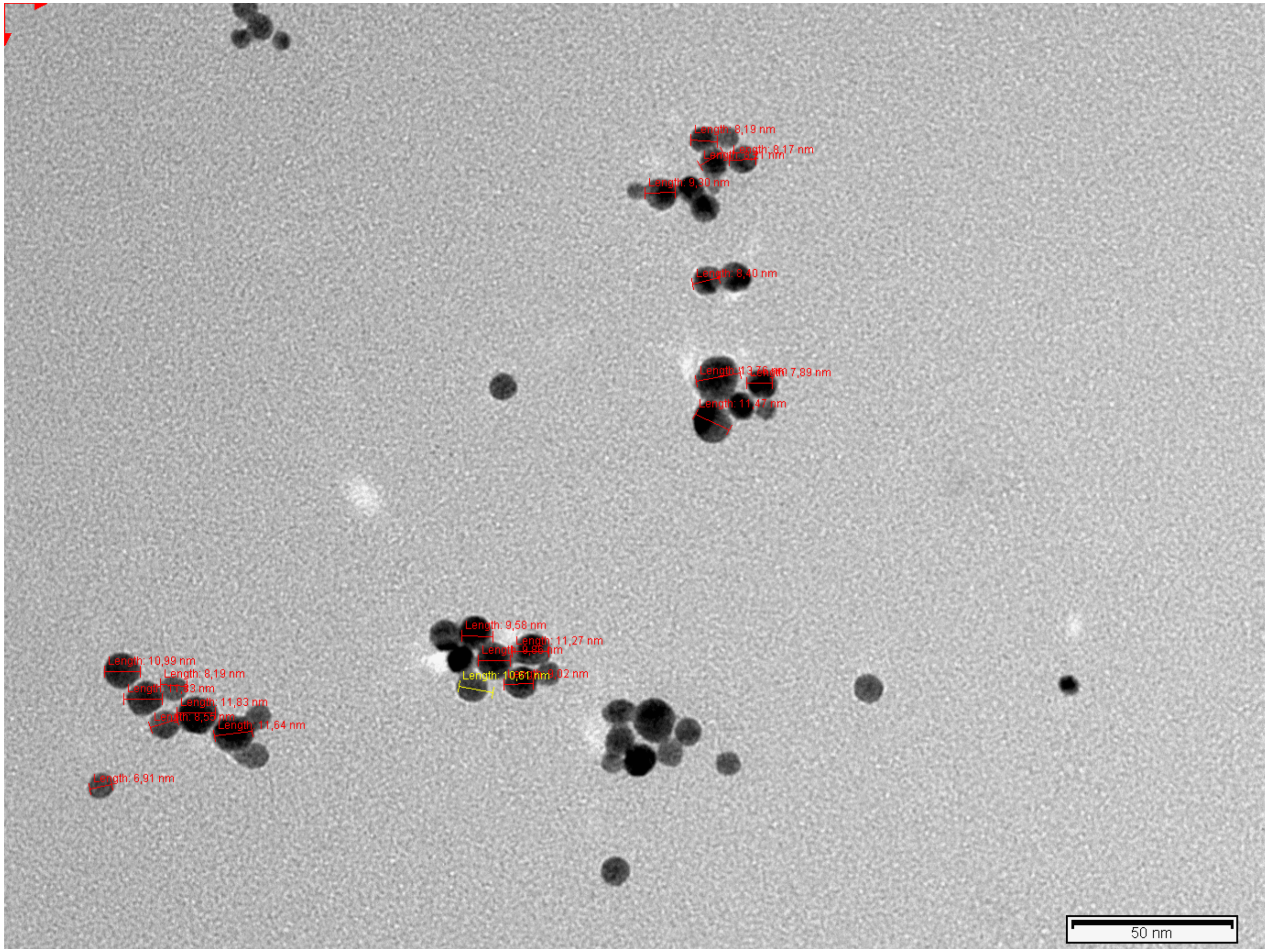
Length: 8,19 nm
Length: 8,17 nm
Length: 9,30 nm
Length: 8,40 nm
Length: 11,47 nm
Length: 9,58 nm
Length: 11,27 nm
Length: 10,61 nm
Length: 10,99 nm
Length: 8,19 nm
Length: 11,83 nm
Length: 6,91 nm
50 nm

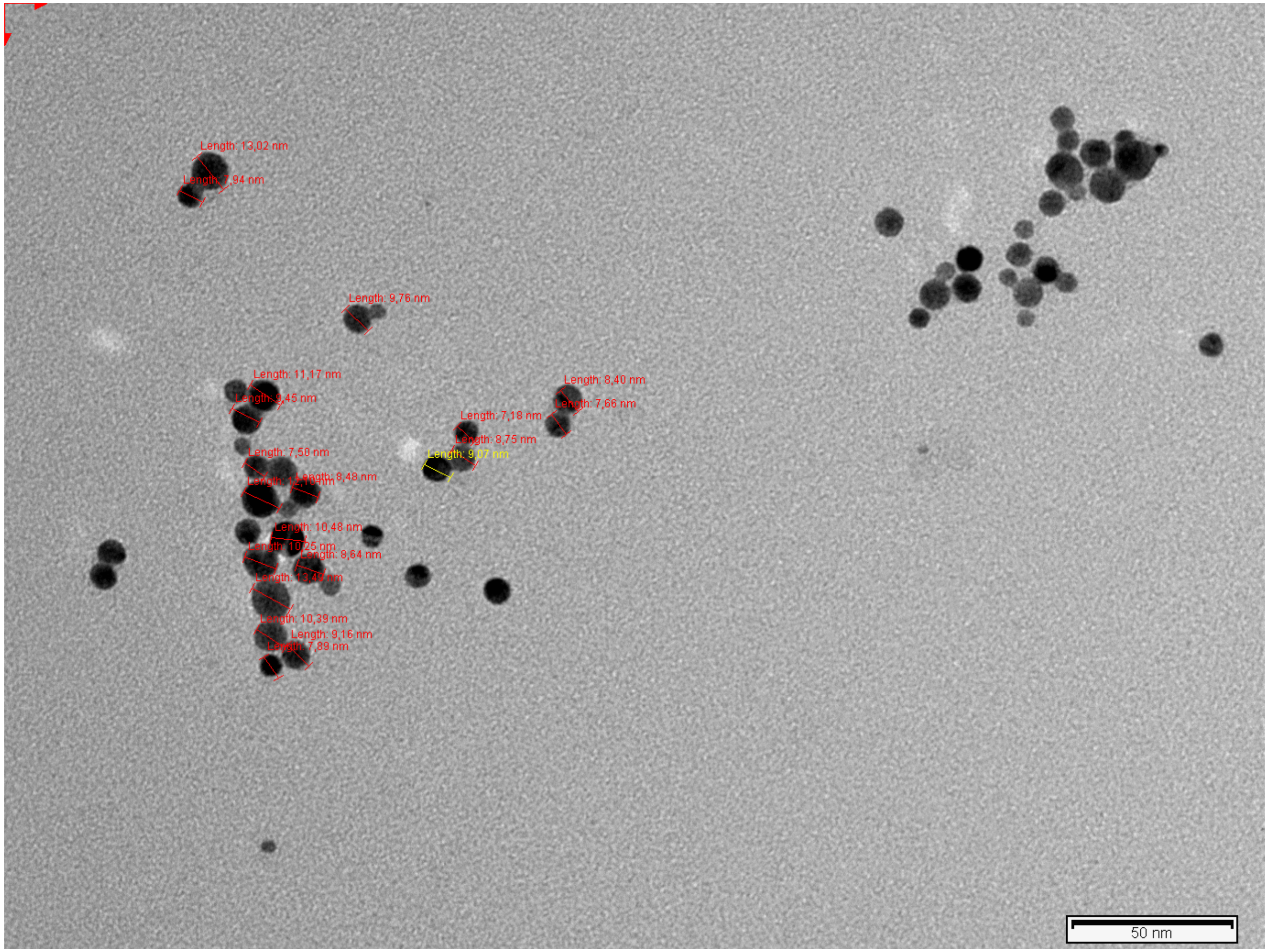

Length: 13,02 nm
Length: 7,94 nm
Length: 9,76 nm
Length: 11,17 nm
Length: 9,45 nm
Length: 8,40 nm
Length: 7,66 nm
Length: 7,18 nm
Length: 8,75 nm
Length: 9,07 nm
Length: 7,50 nm
Length: 12,10 nm
Length: 8,48 nm
Length: 10,48 nm
Length: 10,25 nm
Length: 8,64 nm
Length: 13,49 nm
Length: 10,39 nm
Length: 9,16 nm
Length: 7,89 nm
50 nm

Table S2. TEM image and characterization data of AgNPs@PC-HC (healthy controls) in Milli-Q-water.

**AgNPs@PC-HC (nm)**	
1	11.97
2	12.71
3	12.36
4	9.95
5	10.20
6	13.58
7	12.31
8	14.13
9	11.29
10	12.23
11	8.67
12	12.13
13	11.57
14	11.41
15	13.65
16	12.12
17	12.20
Count	17
Mean	11.91
Minimum	8.67
Maximum	14.13
Standar Deviation	1.37

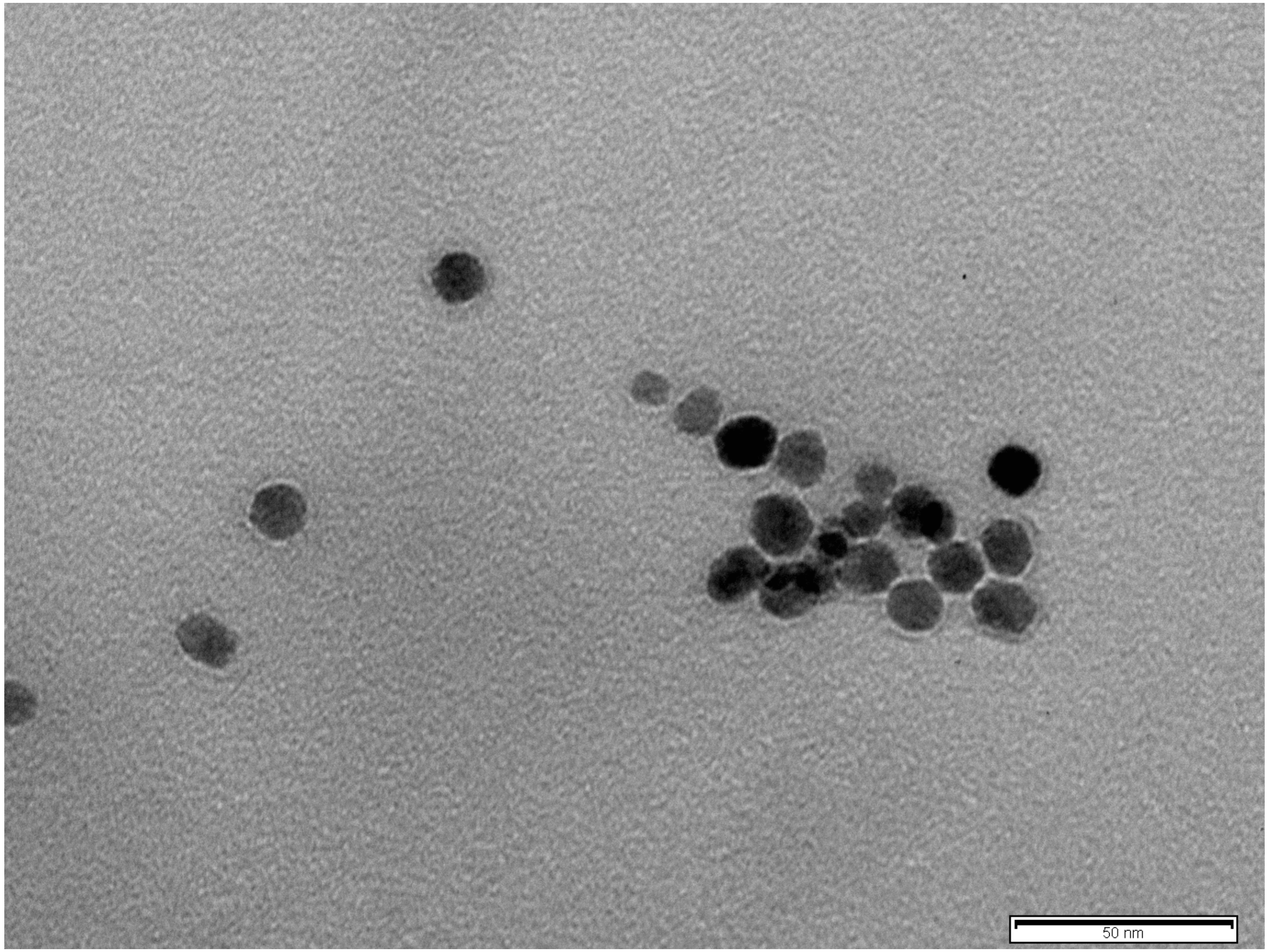
50 nm

Table S3. TEM image and characterization data of AgNPs@PC-NMIBC (patients) in Milli-Q-water.

**AgNPs@PC-NMIBC (nm)**	
1	14.39
2	13.47
3	12.60
4	13.54
5	12.25
6	11.59
7	13.27
8	12.08
9	10.94
10	15.23
11	17.86
Count	11
Mean	13.38
Minimum	10.94
Maximum	17.86
Standar Deviation	1.93

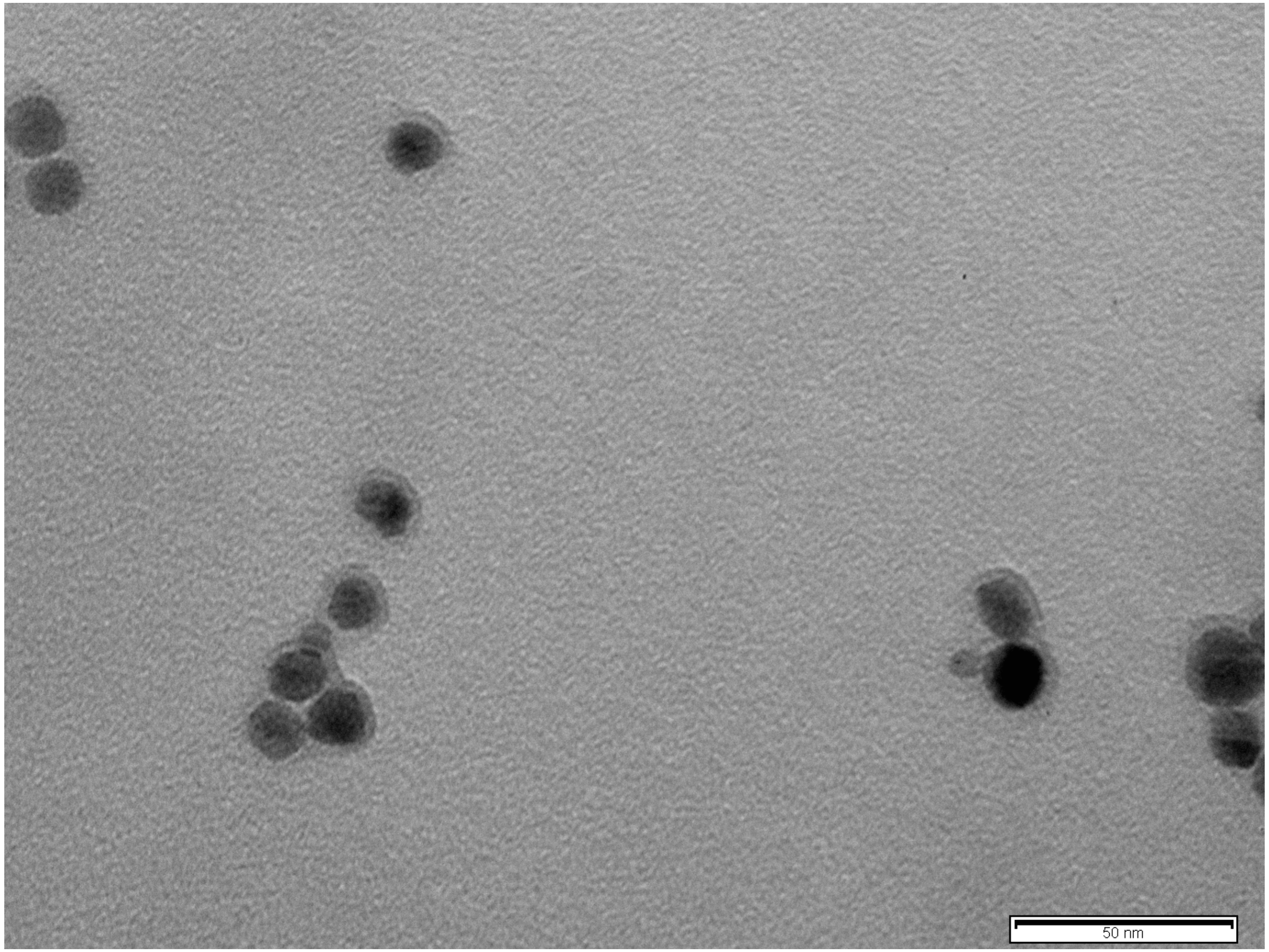
50 nm

Table S4. Differentially expressed proteins (up- and down-regulated) (p-value ≤ 0.05) detected in NMIBC patients (subtypes T1 and Ta) relative to the control group after the analysis of the PC coated AgNPs (9.73 ± 1.70 nm) by SWATH-MS.

	Protein Name	UniProt Name	Gene	*p*-value	Fold Change	
Control *vs.* T1	**Coagulation factor IX**	**FA9_HUMAN**	**F9**	**0,00027325**	**1,556427466**	**↑ T1**
	**Complement C1r subcomponent**	**C1R_HUMAN**	**C1R**	**0,003153549**	**1,353684058**	**↑ T1**
	**Thrombospondin-1**	**TSP1_HUMAN**	**THBS1**	**0,010749764**	**1,373023301**	**↑ T1**
	**Apolipoprotein C-II**	**APOC2_HUMAN**	**APOC2**	**0,014389697**	**1,814038601**	**↑ T1**
	**Plasminogen**	**PLMN_HUMAN**	**PLG**	**0,023399686**	**1,350545683**	**↑ T1**
	**Serum amyloid A-4 protein**	**SAA4_HUMAN**	**SAA4**	**0,028039723**	**1,771595498**	**↑ T1**
	**Plasma kallikrein**	**KLKB1_HUMAN**	**KLKB1**	**0,030943499**	**1,245457438**	**↑ T1**
	Beta-2-glycoprotein 1	APOH_HUMAN	APOH	0,031038566	1,413139568	↑ T1
	**Coagulation factor XII**	**FA12_HUMAN**	**F12**	**0,036056657**	**1,320811796**	**↑ T1**
	**Lipopolysaccharide-binding protein**	**LBP_HUMAN**	**LBP**	**0,038383686**	**1,483385757**	**↑ T1**
	**Complement C1s subcomponent**	**C1S_HUMAN**	**C1S**	**0,040399897**	**1,323391355**	**↑ T1**
	**Carboxypeptidase N catalytic chain**	**CBPN_HUMAN**	**CPN1**	**1,07E-06**	**1,760706885**	**↑ Control**
	**Apolipoprotein M**	**APOM_HUMAN**	**APOM**	**2,49E-05**	**1,878243864**	**↑ Control**
	Complement C4-A	CO4A_HUMAN	C4A	3,35E-05	1,872021358	↑ Control
	Apolipoprotein F	APOF_HUMAN	APOF	0,000241559	2,110329795	↑ Control
	**Insulin-like growth factor-binding protein complex acid labile subunit**	**ALS_HUMAN**	**IGFALS**	**0,000288722**	**1,493728327**	**↑ Control**

	**Carboxypeptidase N subunit 2**	**CPN2_HUMAN**	**CPN2**	**0,000333613**	**1,475909706**	**↑ Control**
	Complement factor B	CFAB_HUMAN	CFB	0,000636998	1,372510381	↑ Control
	**Alpha-2-HS-glycoprotein**	**FETUA_HUMAN**	**AHSG**	**0,001026046**	**1,631445446**	**↑ Control**
	Complement C4-B	CO4B_HUMAN	C4B	0,001425038	1,622969299	↑ Control
	**Fibronectin**	**FINC_HUMAN**	**FN1**	**0,001730097**	**1,657544918**	**↑ Control**
	**Serum paraoxonase/arylesterase 1**	**PON1_HUMAN**	**PON1**	**0,002205149**	**1,510381818**	**↑ Control**
	Complement component C8 gamma chain	CO8G_HUMAN	C8G	0,003262992	1,467525612	↑ Control
	Ficolin-2	FCN2_HUMAN	FCN2	0,003600193	1,765148338	↑ Control
	Apolipoprotein E	APOE_HUMAN	APOE	0,004492395	1,342803692	↑ Control
	Apolipoprotein A-IV	APOA4_HUMAN	APOA4	0,004832934	1,330273828	↑ Control
	Hyaluronan-binding protein 2	HABP2_HUMAN	HABP2	0,005064575	1,385807696	↑ Control
	**Antithrombin-III**	**ANT3_HUMAN**	**SERPINC1**	**0,005098494**	**1,365268921**	**↑ Control**
	**Mannan-binding lectin serine protease 1**	**MASP1_HUMAN**	**MASP1**	**0,006757744**	**1,42660064**	**↑ Control**
	Platelet basic protein	CXCL7_HUMAN	PPBP	0,007192858	1,607064055	↑ Control
	**Complement C2**	**CO2_HUMAN**	**C2**	**0,009799227**	**1,331702175**	**↑ Control**
	**Sex hormone-binding globulin**	**SHBG_HUMAN**	**SHBG**	**0,010572352**	**1,694430151**	**↑ Control**
	Pigment epithelium-derived factor	PEDF_HUMAN	SERPINF1	0,0177288	2,054793133	↑ Control
	**Prothrombin**	**THRB_HUMAN**	**F2**	**0,018741921**	**1,269520963**	**↑ Control**
	**N-acetylmuramoyl-L-alanine amidase**	**PGRP2_HUMAN**	**PGLYRP2**	**0,01966924**	**1,448722587**	**↑ Control**
	Complement C5	CO5_HUMAN	C5	0,022678206	1,261047319	↑ Control

	Vitamin K-dependent protein C	PROC_HUMAN	PROC	0,023388014	1,233676674	↑ Control
	Coagulation factor X	FA10_HUMAN	F10	0,028581041	1,261414143	↑ Control
	Apolipoprotein A-II	APOA2_HUMAN	APOA2	0,029458523	2,388772729	↑ Control
	**Plasma protease C1 inhibitor**	**IC1_HUMAN**	**SERPING1**	**0,044659515**	**1,307368825**	**↑ Control**
**Control *vs.* Ta**	**Protein Name**	**UniProt Name**		***p*-value**	**Fold Change**	
	**Coagulation factor IX**	**FA9_HUMAN**	**F9**	**1,11E-06**	**1,623429779**	**↑ Ta**
	**Thrombospondin-1**	**TSP1_HUMAN**	**THBS1**	**1,25E-06**	**1,582147778**	**↑ Ta**
	Fibrinogen alpha chain	FIBA_HUMAN	FGA	0,00018616	1,692142233	↑ Ta
	**Complement C1s subcomponent**	**C1S_HUMAN**	**C1S**	**0,0006224**	**1,500323307**	**↑ Ta**
	Complement component C7	CO7_HUMAN	C7	0,0014036	1,37740437	↑ Ta
	Vitronectin	VTNC_HUMAN	VTN	0,00148246	1,330829684	↑ Ta
	**Plasminogen**	**PLMN_HUMAN**	**PLG**	**0,00258423**	**1,357256198**	**↑ Ta**
	Complement component C9	CO9_HUMAN	C9	0,00259827	1,326995622	↑ Ta
	**Coagulation factor XII**	**FA12_HUMAN**	**F12**	**0,00302159**	**1,391945692**	**↑ Ta**
	**Complement C1r subcomponent**	**C1R_HUMAN**	**C1R**	**0,00303922**	**1,280967713**	**↑ Ta**
	Vitamin D-binding protein	VTDB_HUMAN	GC	0,003928	1,340366003	↑ Ta
	Complement component C6	CO6_HUMAN	C6	0,0042717	1,446886498	↑ Ta
	Complement component C8 beta chain	CO8B_HUMAN	C8B	0,00538557	1,255996294	↑ Ta
	Hemoglobin subunit alpha	HBA_HUMAN	HBA1	0,00590282	5,020407249	↑ Ta
	**Lipopolysaccharide-binding protein**	**LBP_HUMAN**	**LBP**	**0,00680537**	**1,634749119**	**↑ Ta**
	Galectin-3-binding protein	LG3BP_HUMAN	LGALS3BP	0,00753614	2,016590504	↑ Ta
	Inter-alpha-trypsin inhibitor heavy chain H1	ITIH1_HUMAN	ITIH1	0,00788349	0,719608487	↑ Ta

	Complement factor I	CFAI_HUMAN	CFI	0,00810041	1,222227898	↑ Ta
	Immunoglobulin lambda variable 3-27	LV327_HUMAN	IGLV3-27	0,01005932	1,783180848	↑ Ta
	Hemoglobin subunit beta	HBB_HUMAN	HBB	0,01030231	4,673494773	↑ Ta
	**Serum amyloid A-4 protein**	**SAA4_HUMAN**	**SAA4**	**0,01145609**	**2,751315105**	**↑ Ta**
	**Apolipoprotein C-II**	**APOC2_HUMAN**	**APOC2**	**0,01459098**	**2,018908776**	**↑ Ta**
	Alpha-1-antichymotrypsin	AACT_HUMAN	SERPINA3	0,01546691	5,505121842	↑ Ta
	C4b-binding protein alpha chain	C4BPA_HUMAN	C4BPA	0,01766023	1,267495989	↑ Ta
	Alpha-1B-glycoprotein	A1BG_HUMAN	A1BG	0,03046776	1,640307834	↑ Ta
	Alpha-1-antitrypsin	A1AT_HUMAN	SERPINA1	0,03633694	3,239474829	↑ Ta
	Ceruloplasmin	CERU_HUMAN	CP	0,03816982	1,652775639	↑ Ta
	Immunoglobulin heavy variable 3-30-5	HV335_HUMAN	IGHV3-35	0,04091296	1,749806744	↑ Ta
	**Plasma kallikrein**	**KLKB1_HUMAN**	**KLKB1**	**0,04413498**	**1,172777632**	**↑ Ta**
	Complement factor H	CFAH_HUMAN	CFH	0,04493203	1,213794163	↑ Ta
	**Carboxypeptidase N catalytic chain**	**CBPN_HUMAN**	**CPN1**	**9,52E-07**	**1,569696866**	**↑ Control**
	**Apolipoprotein M**	**APOM_HUMAN**	**APOM**	**9,18E-06**	**1,65564258**	**↑ Control**
	**Carboxypeptidase N subunit 2**	**CPN2_HUMAN**	**CPN2**	**1,14E-05**	**1,479498919**	**↑ Control**
	**Alpha-2-HS-glycoprotein**	**FETUA_HUMAN**	**AHSG**	**3,36E-05**	**1,672329927**	**↑ Control**
	**Antithrombin-III**	**ANT3_HUMAN**	**SERPINC1**	**0,00012924**	**1,410236969**	**↑ Control**
	**N-acetylmuramoyl-L-alanine amidase**	**PGRP2_HUMAN**	**PGLYRP2**	**0,00046273**	**1,601708127**	**↑ Control**
	**Mannan-binding lectin serine protease 1**	**MASP1_HUMAN**	**MASP1**	**0,0005914**	**1,440252665**	**↑ Control**

	**Serum paraoxonase/arylesterase 1**	**PON1_HUMAN**	**PON1**	**0,00144113**	**1,402785174**	**↑ Control**
	**Prothrombin**	**THRB_HUMAN**	**F2**	**0,00196398**	**1,299723373**	**↑ Control**
	**Insulin-like growth factor-binding protein complex acid labile subunit**	**ALS_HUMAN**	**IGFALS**	**0,00282756**	**1,291383968**	**↑ Control**
	Vitamin K-dependent protein S	PROS_HUMAN	PROS1	0,00653335	1,241725216	↑ Control
	Alpha-2-antiplasmin	A2AP_HUMAN	SERPINF2	0,00979701	1,476811908	↑ Control
	**Fibronectin**	**FINC_HUMAN**	**FN1**	**0,01079141**	**1,357224248**	**↑ Control**
	**Plasma protease C1 inhibitor**	**IC1_HUMAN**	**SERPING1**	**0,01147388**	**1,316405301**	**↑ Control**
	**Complement C2**	**CO2_HUMAN**	**C2**	**0,01487246**	**1,228505056**	**↑ Control**
	**Sex hormone-binding globulin**	**SHBG_HUMAN**	**SHBG**	**0,03363129**	**1,440623383**	**↑ Control**
